# First Two Cases of Fungal Infections Associated with Multi-drug Resistant Yeast, *Fereydounia khargensis*

**DOI:** 10.1007/s11046-016-0002-y

**Published:** 2016-03-24

**Authors:** Ratna Mohd Tap, Nur Yasmin Ramli, Parameswari Sabaratnam, Rohaidah Hashim, Ahmed Rafezzan Ahmed Bakri, Lim Bee Bee, Stephanie Jane Ginsapu, Rahimah Ahmad, Mohd Fuat Abd Razak, Norazah Ahmad

**Affiliations:** Bacteriology Unit, Infectious Diseases Research Centre, Institute for Medical Research, 50588 Jalan Pahang, Kuala Lumpur, Malaysia; Microbiology Unit, Pathology Department, Queen Elizabeth Hospital, 88200 Kota Kinabalu, Sabah Malaysia; Microbiology Unit, Pathology Department, Sultanah Fatimah Specialist Hospital, 84000 Muar, Johor Malaysia; Haematology Unit, Cancer Research Centre, Institute for Medical Research, Jalan Pahang, Kuala Lumpur, 50588 Malaysia

**Keywords:** *Fereydounia khargensis*, Urocystidales, Ustilaginomycotina, Multi-drug resistance

## Abstract

The number of new fungal pathogens is increasing due to growing population of immunocompromised patients and advanced identification techniques. *Fereydounia khargensis* is a yeast and was first described in 2014 from environmental samples. As far as we know, this is the first report of human infections associated with *F. khargensis*. The yeasts were isolated from blood of a HIV-positive patient and pleural fluid of chronic renal failure patient. Amplification and sequencing of the internal transcribed spacer and the large subunit regions confirmed the identity of the isolates. Both isolates showed multi-drug resistance to antifungal agents tested.

## Introduction

*Fereydounia khargensis* was first described by Nasr et al. [1], isolated from plant remnants on the Kharg Island in the Persian Gulf of Iran. Based on phylogenetic analysis of the large subunit (LSU) in the ribosomal RNA (rRNA) gene, the organism is clustered in the same order (Urocystidales) with a few species of smut fungi, for example *Ustacystis waldsteiniae*, *Mandkurella kalopanacis*, *Doassansiopsis deformans* and *Thecaphora seminis*-*convolvuli* [1]. *F*. *khargensis* represents a new lineage in the Urocystidales, of the subphylum Ustilaginomycotina. Members of Ustilaginomycotina are usually dimorphic, producing a saprobic haploid yeast phase and a parasitic dikaryotic hyphal phase [2]. There are about 1500 species of subphylum Ustilaginomycotina [2, 3], and they are found largely in temperate climate countries rather than in tropical countries [4]. Established as plant parasites, few species of Ustilaginomycotina had been reported to cause infection in human, i.e. *Pseudozyma antarctica*, *P. parantarctica*, *P. thailandica* and *P. aphidis* [5–7].

The identification of *F. khargensis* by conventional phenotypic methods is almost impossible, due to unfamiliar characteristics of the isolates. Therefore, the identification was accomplished by amplification and sequencing of the internal transcribed spacer (ITS) and LSU regions in the rRNA gene. We report the first two cases of *F. khargensis* isolated from the blood sample of a human immunodeficiency virus (HIV)-positive patient and the pleural fluid of a chronic renal failure patient. In vitro susceptibility testing against amphotericin B, caspofungin, anidulafungin, fluconazole, itraconazole and voriconazole was also carried out on the yeast isolates.

## Case Studies

### Case 1

A 33-year-old HIV-positive patient was admitted to Queen Elizabeth Hospital in Kota Kinabalu, Sabah, Malaysia, due to developed episodes of fever associated with chills and rigours that persisted for 2 weeks. The fever was high grade, ranging between 39 and 40 °C, and was persistent in nature. There were no other associated symptoms such as cough, shortness of breath, chest pain, abdominal pain or diarrhoea. The patient did not suffer from headache or visual disturbance. There was no loss of appetite; however, the patient had weight loss. Apart from the high temperature recorded, general physical examination was unremarkable. Further investigation such as chest X-ray did not revealed the cause of the fever. On admission, his HIV viral load was 2.4 × 10^5^ copies with a CD4 count of 63 cells/mm^3^. However, the viral load decreased to <20 copies after 5 days of highly active antiretroviral therapy (HAART) and his CD4 count improved to 353 cells/mm^3^ 9 days after the initial reading. The patient was empirically started on intravenous (IV) ceftriaxone 1 g three times/day, oral co-trimoxazole 160 mg/800 mg daily and IV Tazocin 4.5 g every 8 h.

The blood culture that was performed on the day of admission did not reveal any growth. It was repeated on several occasions, positive on sixth day of hospitalisation after 48 h of incubation. The Gram stain of the positive blood culture revealed yeast elements. Following the blood culture finding, amphotericin B was given with initial IV infusion of 1–5 mg/day of amphotericin B, gradually increasing it to 0.4–0.6 mg/kg daily for 7 days. Treatment was switched to IV itraconazole 200 mg twice daily for 2 days, followed by 200 mg/day for 12 days as the patient did not improve clinically after treatment with amphotericin B. The patient’s clinical condition markedly improved after 5 days following this new antifungal regime.

### Case 2

A 59-year-old male, with a known case of end-stage renal failure on continuous ambulatory peritoneal dialysis (CAPD), hypertension, diabetes mellitus and hepatitis B carrier, was admitted to Sultanah Fatimah Hospital in Muar, Johor, Malaysia, due to a dislodged distal connector of Tenckhoff catheter after an alleged fall in the toilet at home. There was no loss of consciousness, but he had nausea and dizziness prior to fall. After the event, the dislodged connector was reconnected to the Tenckhoff catheter and used for CAPD. There was no history of abdominal pain, distention or fever.

On examination, the patient was conscious and alert. The lungs were clear, and the abdomen was soft and non-tender. Daily full examination and microscopy examination (FEME) and cell count of the peritoneal fluid (PF) presented clear and colourless, with no white blood cell (WBC) seen until the fifth day of the admission. On the fifth day, the FEME of the PF revealed 30 WBC/µl, with 90 % polymorphs and 10 % lymphocytes. On the sixth day, the WBC count of the PF increased to a significant level, i.e. 225 WBC/µl with 80 % polymorphs and 20 % lymphocytes indicative of an infection. It was sent for a culture in aerobic blood culture bottle. After 48 h incubation, Gram stain exhibited a mixed growth of Gram-negative, rod-shaped bacterium and yeast-like cells. The Gram-negative bacteria were identified as *Pseudomonas fluorescens;* however, the yeast-like organism was unable to be identified using the ID 32C yeast identification systems. No specimen was sent in anaerobic blood culture bottle. A repeat PF culture was sent in aerobic blood culture bottle on the 9 days which grew solely a yeast-like organism with the same characteristics as the fifth day. The PF was also sent for culture in anaerobic blood culture bottle on the eighth day and it grew coagulase-negative methicillin-resistant *Staphylococcus*.

A dosage of 400 mg/day once a day of fluconazole was started following the yeast growth from the PF. The patient’s Tenckhoff catheter was removed immediately and changed to a new one following the positive PF culture. The clinical condition of the patient improved after 10 days of the fluconazole treatment and he was discharged well. On the subsequent follow-up 1 month later, his PF cell count was reducing in trend. His PF WBC count was 75 WBC/µl with 30 % polymorphs and 70 % lymphocytes.

## Mycological Investigations

**Fig. 1:**
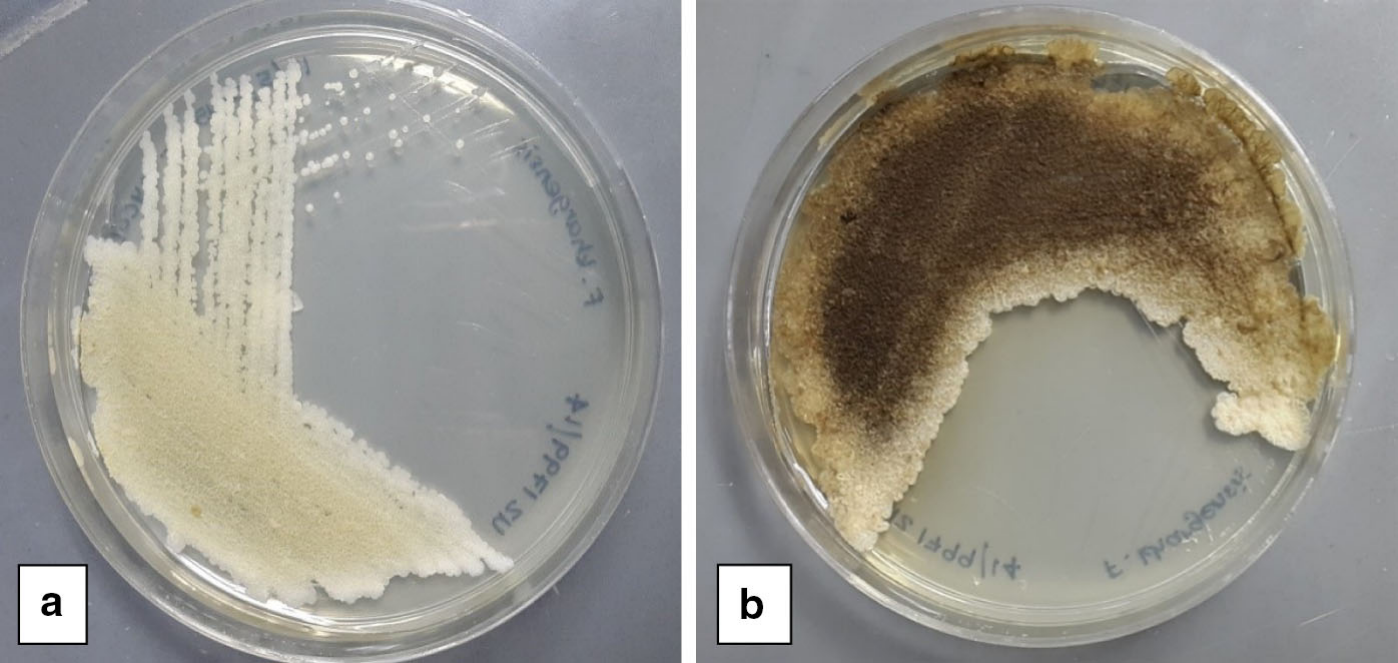
Yeast-like colonies of strain UZ1799/14 on SDA plate after incubation at 48 h (**a**) and the melanin-like pigment was clearly seen after 120 h incubation (**b**)

Both positive cultures grew yeast-like organisms on Sabouraud’s dextrose agar (SDA) and were sent to the Mycology Laboratory at the Institute for Medical Research (IMR) for identification. Upon receival at the laboratory, the yeast-like colonies were subcultured onto new SDA plates and incubated at 35 °C for further tests. The isolates of Case 1 and Case 2 were designated as strain UZ1799/14 and UZ1801/15, respectively. Growths on both SDA plates represented as cream coloured yeast-like colonies, dry and with slightly wrinkled and fringed margins (Fig. 1a). However, after 72 h of incubation, the colonies for both strains started producing melanin-like pigmentation and it became even darker after 120 h of incubation (Fig. 1b).

**Fig. 2:**
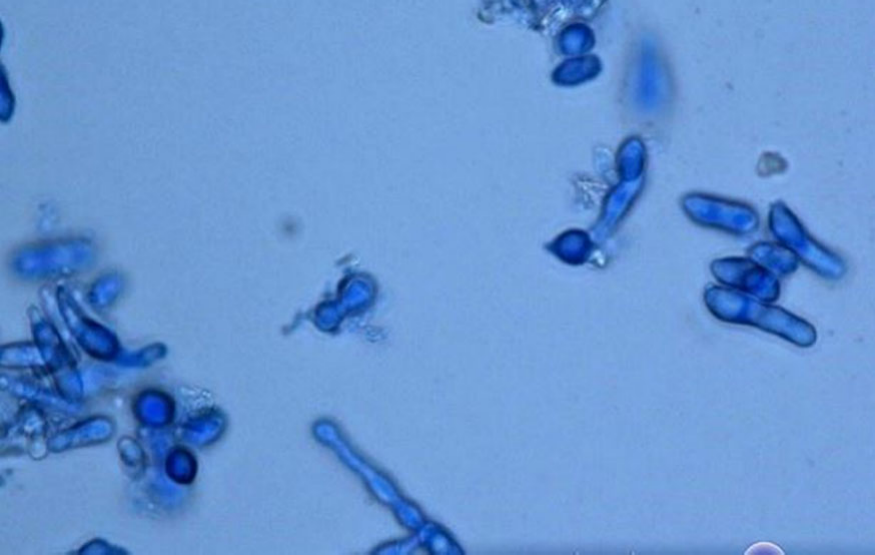
LPCB stain of *F. khargensis* at ×100 magnification showing bipolar budding of blastospores

**Fig. 3:**
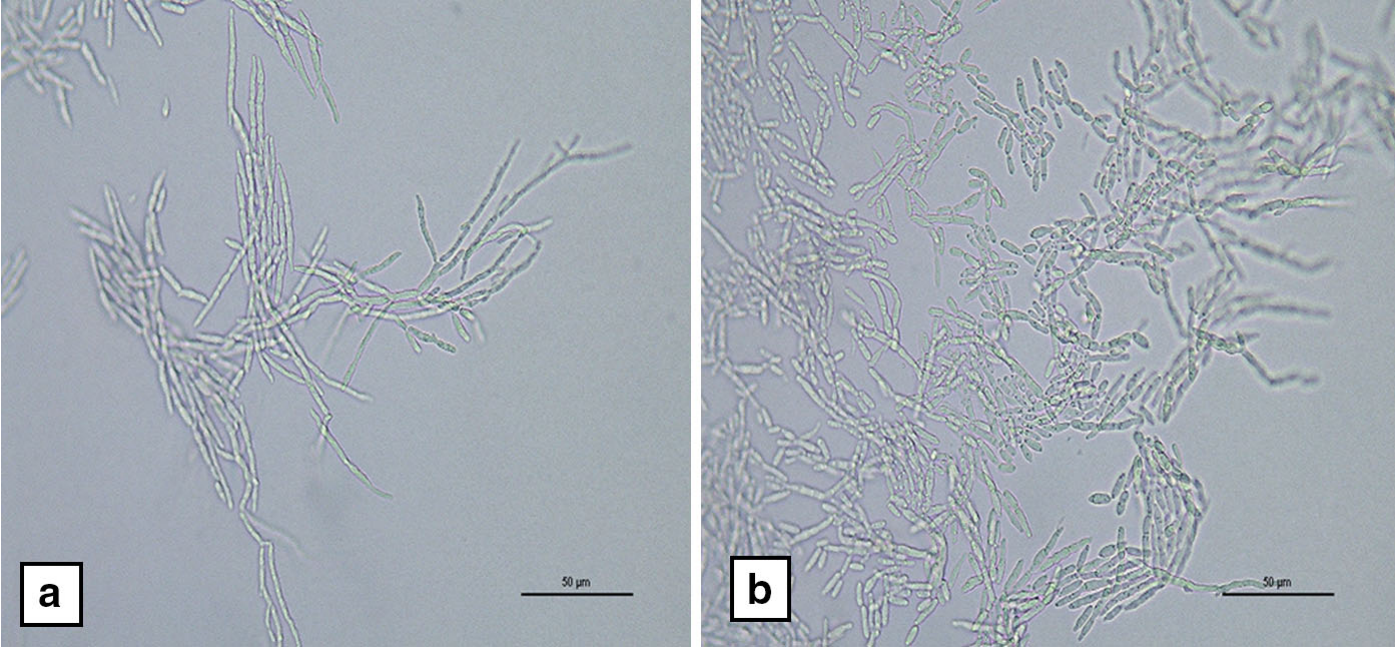
Production of branching hyphae (**a**); pseudohyphae and blastospores (**b**) on corn meal agar of the slide cultures, magnification ×40

Lactophenol cotton blue (LPCB) smear of 48-h-old isolates revealed short stalk reproduced by polar budding of ellipsoidal and elongation of blastospore (Fig. 2). Slide cultures were also carried out on corn meal agar and incubated at room temperature (25 °C) for observation of hyphae production. After 72 h of incubation, the slide cultures revealed production of hyphae, pseudohyphae and blastospores (Fig. 3).

Assimilation tests were carried out by API 20C AUX (bioMe´rieux, Marcy l’Etoile, France) and VITEK 2 system (bioMe´rieux, Marcy l’Etoile, France) following the manufacturer protocols. Both systems misidentified the isolates, in which the API 20C and VITEK 2 produced results as *Cryptococcus neoformans* (98 % probability) and *Cryptococcus laurentii* (89 % probability), respectively.

## Identification by Molecular Methods

DNA extractions, amplifications by polymerase chain reaction (PCR), PCR products purification and sequencing methods were performed as previously described [8]. The internal transcribed spacer (ITS) and the large subunit (LSU) in rRNA gene regions were amplified using universal primers ITS5/ITS4 and NL1/NL4 as previously described by Scholch et al. [9] and O’Donnell [10], respectively. NCBI BLAST search (http://blast.ncbi.nlm.nih.gov/) was performed for the ITS and LSU sequences from both isolates; resulting in a 99.7 and 100.0 % matched to the reference strain, IBRC-M30116 (Table 1).

**Table 1:** Sources, accession numbers and nucleotides similarity between the three strains of *F. khargensis*

Strain name	Sample source	Origin country	ITS similarity (%)	ITS GenBank accession no.	LSU similarity (%)	LSU GenBank accession no.	Reference
UZ1799/14	Human blood	Malaysia	99.7	KP326581	100.0	KT362712	This report
UZ1801/15	Human pleural fluid	Malaysia		KU212883		KU321687	This report
IBRC-M30116	Plant remnants	Iran		KJ490642		KJ490641	[1]

## In Vitro Susceptibility Test

In vitro susceptibility test was carried out using the *E* test method. The method was performed according to the manufacturer’s protocols (AB Biodisk 2007). The antifungals tested were amphotericin B, caspofungin, anidulafungin, fluconazole, itraconazole and voriconazole. The minimum inhibitory concentration (MIC) of the antifungals was determined at 24 and 48 h (Table 2) and the interpretation of the MIC followed the Clinical and Laboratory Standards Institute (CLSI) guidelines (2008). In vitro susceptibility pattern showed that fluconazole, itraconazole and voriconazole have good activity against the yeast, although the MIC readings of the azole agents for strain UZ1801/15 were higher than UZ1799/14. On the other hand, both isolates showed resistant pattern to amphotericin B, caspofungin and anidulafungin with MIC reading of >32.000 µg ml^−1^ for amphotericin B, caspofungin and anidulafungin; except the MIC for caspofungin of the strain UZ1801/15 which was 4.000 µg ml^−1^.

**Table 2:** MICs and susceptibility interpretations of the isolates against antifungals

	MIC (µg ml^-1^)			
	Strain UZ1799/14		Strain UZ1801/15	
	24 h	48 h	24 h	48 h
Amphotericin B	>32.000	>32.000 (R)	16.000	>32.000 (R)
Caspofungin	>32.000	>32.000 (R)	2.000	4.000 (R)
Anidulafungin	>32.000	>32.000 (R)	>32.000	>32.000 (R)
Fluconazole	0.500	2.000 (S)	3.000	8.000 (S)
Itraconazole	0.047	0.094 (S)	0.094	0.125 (S)
Voriconazole	0.008	0.032 (S)	1.000	2.000 (S)

*S* sensitive, *R* resistant

## Discussion

We report to our knowledge, the first two cases of fungal infection associated with *F. khargensis* in immunocompromised patients. The isolates produced melanin-like pigmentation on SDA and were accurately identified based on amplification of the ITS and LSU regions. In vitro susceptibility test of the isolates showed multi-drug resistance against amphotericin B, anidulafungin and caspofungin, but sensitive to azole agents tested.

*Fereydounia khargensis* characterises a new lineage in the Urocystidales of the subphylum Ustilaginomycotina. In this case report, the organism exists as yeast phase on normal isolation media, i.e. Sabouraud’s dextrose agar (SDA), and as hyphal phase on corn meal agar. It grew well at 35 °C and the growth rate is quite fast (within 24 h). The initial colony characteristics are more similar to *Trichosporon* spp. rather than *Candida* spp., i.e. cream coloured, dry and slightly wrinkled. However, the production of melanin-like pigmentation after 3 days incubation on SDA is a unique characteristic of the isolate. However, the same species strain IBRC-M30116 do not produce melanin-like pigmentation after 7 days incubation on yeast peptone glucose (YPG), malt extract agar (MEA), malt yeast 40 % sucrose agar (M40Y) and dichloran 18 % glycerol (DG18) at 28 °C [1].

Conventional identification methods did not allow the accurate identification of *F. khargensis*. The microscopic characteristics of *F*. *khargensis* from LPCB staining and slide culture were unfamiliar to us. In addition, the commercial yeast identification systems (API 20C and VITEK 2) were also unable to identify the isolate due to limited databases of the systems. Therefore, diagnostic laboratories in which the yeast identification systems depend on the colony morphology and commercial system may not be able to accurately identify this new and uncommon yeast. Fortunately with the molecular methods available, the identification was made possible. The two regions in the rRNA gene, i.e. ITS and LSU, are variable and have been used for identification of many uncommon yeast spp. [7, 11–13].

In this case report, the low CD4 count of Case 1 patient and the complicated medical conditions of Case 2 patient predisposed the patients to immunodeficiency, thus susceptible to the infections of *F. khargensis*. The interactions between host immune status and pathogen have been reported to be the crucial determinants of the type of invasive fungal infection among patients at risk of it [14]. For example, there are many ascomycetous yeast species from the *Candida* genus that can become pathogens when the host’s resistance to infection is locally or systemically impaired. In an immunocompromised host, translocation from the gastrointestinal tract and intravascular catheters are the two main portals of entry for disseminated yeast infection [14, 15].

Antifungal treatment remains a challenge for patient with unusual yeast infection, since to date only a few antifungal agents are currently available. The situation is compounded when most available antifungal drugs are usually associated with serious side effects and the fact that some organisms have developed resistance against antifungals [16]. In this case report, findings of in vitro susceptibility test were parallel with in vivo. For instance, patient 1 was initially treated with amphotericin B; however, his clinical condition was not improved until it was switched to itraconazole, parallel with our in vitro study that showed the isolate to be resistant to amphotericin B, but sensitive to itraconazole. In patient 2, the clinical condition was improved after 1 week of fluconazole treatment. A similar finding was observed in vitro, in which the isolate was sensitive to azole agents tested (fluconazole, itraconazole and voriconazole).

With the development in identification methods and the increasing population of patients who are immunodeficient or undergoing long-term immunosuppression treatment, the number of new and uncommon yeasts that cause diseases is growing. Thus, it has become a challenge to accurately identify these species. The growing number of resistant yeast species has also simultaneously increased, thus posing a challenge in antifungal treatment and patient management. Pathogenicity, mode of transmission and resistance mechanism of *F.khargensis* remain unanswered. Further investigation of *F.khargensis* is necessary to accomplish the gaps.

## Conclusion

This study reported *F.khargensis* causing septicaemia in immunocompromised patients. Commercially available systems failed to identify the yeasts. However, with the advancement of technology, the isolates were correctly identified by PCR-sequencing, i.e. by amplification of the ITS and LSU region of the rRNA gene.
